# Using the Hidden Curriculum to Teach Professionalism in Nursing Students

**DOI:** 10.5812/ircmj.15532

**Published:** 2014-03-05

**Authors:** Zohreh Karimi, Tahereh Ashktorab, Easa Mohammadi, Heidar Ali Abedi

**Affiliations:** 1 Department of Nursing, Nursing and Midwifery School, Ahvaz Jundishapur University of Medical Sciences, Ahvaz, IR Iran; 2 Department of Nursing, Nursing and Midwifery School, Shahid Beheshti University of Medical Sciences, Tehran, IR Iran; 3 Department of Nursing, Tarbiat Modares University, Tehran, IR Iran; 4 Department of Nursing, Khorasgan Branch, Islamic Azad University, Isfahan, IR Iran

**Keywords:** Curriculum, Education, Nursing, Students, Nursing

## Abstract

**Background:**

Professionalism in nursing is critical for creating credibility and a positive image.

**Objectives:**

This study was carried out to explain the use of hidden curriculum in teaching professionalism in nursing students.

**Materials and Methods:**

This qualitative study was conducted through purposeful sampling strategy by the participation of 32 nursing students. The data were collected by using semi-structured interviews, and this process was continued until achieving data saturation and themes’ emergence. Content analysis method was used for data analysis.

**Results:**

Data analysis revealed three main themes: Development of understanding the professionalism elements, Variety of influenceability strategies, and Influenceability to various resources. Each theme consisted of some subthemes.

**Conclusions:**

The nursing students learnt the professionalism elements by different methods from different resources through the hidden curriculum. Therefore, exploration of the currently administered hidden curricula is suggested.

## 1. Background

Nursing is a profession, and fulfills all prerequisites of a profession (1). It plays an essential role in patient care; however, the nature and the quality of performing this role depend on the preparation of the individual for such performance (i.e. the nursing education), often administered within a four-year academic period. The purpose of nursing education is to educate students who acquire knowledge, practical skills and social responsibility required for accepting their roles as professional nurses (2). Professionalism emphasizes the values and obligation in the provision of services to the society (3). Professionalism in nursing is critical for creating credibility and a positive image of the profession (4). 

Professionalism is the traditional part of a hidden curriculum, understood and caught rather than being taught. And as an outcome, it is implicit, not explicit (5). The implicit curriculum is as important as the explicit one in the formation of professional features (6). Therefore, it is important to reveal the hidden curriculum to improve professionalism. Nevertheless, it is not readily accessible (7). From professional point of view, professionalism is reinforced and stabilized during the time and in interaction with others. The review of literature shows that there are deep social bonds among the students in the Asian countries as compared to the West. Therefore, the influence of the hidden curriculum on the Asian students is higher than that on the Western students (8).

In Iran, students study nursing in the higher education from the Baccalaureate's degree to PhD. A Baccalaureate program is the main nursing program at university level, which should be attended to develop into a professionally registered nurse. The learning environment is shared in the Baccalaureate program via classroom, hospital, community and other educational settings (9). Learning occurs in both classroom and clinical environments, and the nursing students learn professionalism. Hence, the first and the most sensitive stage of professionalism is when the students enter the educational and clinical environments. The description of professionalism helps to find the ways to the development of profession and professional roles, and thus increase professional motivation and improve the provision of care to people. In addition, by nursing education, it is possible to prepare the students to enter into the real professional world more adequately. In spite of the fact that professionalism is under consideration, there is a dearth of materials on learning professionalism through the hidden curriculum in nursing.

The messages and values conveyed by the hidden curriculum, are culturally influenced (10). The qualitative methods are useful for investigating the cultural and contextual aspects of education (11). Considering the importance of the qualitative studies in better understanding a phenomenon, and the fact that qualitative research provides rich information on activities, experiences, events and behaviors, it allows for better description, definition and understanding of activities in the social realm (12). Moreover, qualitative research has naturalistic paradigm (13).

## 2. Objectives

This study aimed to explain using the hidden curriculum to teach professionalism in nursing undergraduates in Iran.

## 3. Materials and Methods

### 3.1. Design

A qualitative design, based on the content analysis approach, was used for data collection and analysis of the perspectives and experiences of nursing students. Qualitative content analysis is the analysis of the content of narrative data to identify prominent themes and patterns among the emerged themes (14).

### 3.2. Setting and Participants

In this study, 32 nursing undergraduates (who were studying in the first to the fourth years of education in the Nursing and Midwifery College, Ahvaz Jundishapur University of Medical Sciences, Iran) were selected through a purposeful sampling strategy. They were chosen by using maximum-variation sampling for the number of years of nursing education to capture a range of perspectives. In this sampling method, the individuals are selected to participate in a qualitative research on their first-hand experience with a culture, social process, or phenomenon of interest (12). According to the objective of the research, characteristics of the participants included: nursing students undergraduates and all of them who announced their desire to participate in the research and to express their own experiences.

### 3.3. Data Collection

This study used face-to-face and semi-structured interviews for data collection by the first author (who is a PhD candidate of nursing). She met all participants, and after describing the aims and methodology of the study, invited them individually to participate in the study. The interviewer accomplished this study after the extensive and thorough study of the qualitative research methods and attending the necessary workshops, as well as enjoying the views of expert teachers in this regard. The main question to begin the interview with was: "Please describe your experiences of professionalism in nursing". Based on the participants' answers, probing questions were asked during the interviews to better understand and clarify their experiences. The interviews lasted for between 60 to 90 minutes. All interviews were tape-recorded, listened and transcribed verbatim. The data collection and analysis were done simultaneously. The content of the interviews was entered into the MAXQDA10 software. The data were gathered in 2012.

### 3.4. Data Analysis

Content analysis was conducted to identify the emerged themes and categories. The following steps were taken to analyze the collected data (15):

1. The interviews were transcribed verbatim and read several times in order to obtain the sense of the whole.
2. The text was divided into meaning units, then condensed.
3. The condensed meaning units were abstracted, and labeled with codes.
4. The codes were sorted into subcategories and categories based on their similarities and differences.
5. Finally, the themes were formulated as the expression of the latent content of the text.

Data collection was continued until achieving data saturation and themes’ emergence. Saturation refers to the repetition of discovered information and confirmation of the previously collected data (12).

### 3.5. Trustworthiness

Trustworthiness was established in accordance with Lincoln and Guba (16) and Graneheim and Lundman (15). Credibility and confirmability were achieved by returning a summary of the interviews to three participants for checking, and confirming that their perspectives were represented. Peer-checking was done by the authors and two doctoral nursing students, which resulted in similar finding. Maximum variation of the sampling enhanced the data credibility and confirmabiliy. The full text of several interviews together with initial coding, concepts and categories was sent to an observer familiar with qualitative research in nursing education, and necessary corrections were made. The data were analyzed independently by the researchers in order to identify and categorize the initial codes. Then the codes and themes were compared.

### 3.6. Ethical Considerations

The research administration was approved by the Ethics Committee of Ahvaz Jundishapur University of Medical Sciences (with the ethical code No. ETH- 522), and the relevant official permits were issued by the Nursing and Midwifery School to perform the sampling and to accomplish the research. Then, based on the research environment, the research aims and methodology were described to the participants, and their informed and written consent was taken regarding their voluntary participation in the research and that they could withdraw from the study at any time. The participants were reassured that their responses and identities would be kept in full confidentiality.

## 4. Results

All of the students approached, agreed to participate in the study, and none of them were turned away. The participants of this study were 32 nursing undergraduates (21 girls and 11 boys) with the age range of 21-26 years old. They were studying in the first to fourth years of their education (Table 1). Data analysis revealed three main themes: 1) Development of understanding the professionalism elements, 2) Variety of influenceability strategies, and 3) Influenceability to various resources. Each theme consisted of different subthemes (Table 2). 

**Table 1.** Demographic Characteristics of the Participants (n = 32) ^a^

Variable	Values
**Age, y**	22.25 ± 1.21
**Gender**	
Male	11 (34.4)
Female	21 (65.6)
**Year of nursing education**	
1	2 (6.3)
2	3 (9.4)
3	15 (46.9)
4	12 (37.5)

^a^ Data are presented as mean ± SD or No. (%)

**Table 2.** Summary of the Findings

Themes
**Development of understanding the professionalism elements**
Learning the professional ethics
Learning patient-centeredness
**Variety of influenceability strategies**
Observational learning
Learning from feedback
Inverse learning
**Influenceability to various resources**
Influenceability to nurse educators
Influenceability to nurses and head nurses
Influenceability to physicians
Influenceability to peers
Influenceability to patients

### 4.1. Development of Understanding the Professionalism Elements

This theme represents the nursing students' views on the main elements of professionalism. In other words, it is their description of professionalism and an account of their learning of these elements. This theme incorporates two subthemes including "learning professional ethics", and "learning patient-centeredness". In fact, students view these subthemes as the elements of professionalism.

#### 4.1.1. Learning Professional Ethics

"Learning professional ethics" was one of the elements of professionalism from the nursing students' view. They noticed this element in the process of learning through the hidden curriculum and tried to internalize it. The development of professional ethics or the ethical aspects of care is considered by the students to include factors like "patience", "humility", and "altruism". Patience was one of the factors which many of the participants addressed in different ways:

"You know, given the short period of time we have, we may not be able to do the required care. Therefore, if we have a little bit of patience and try to do our best, we can actually witness the result both in ourselves and in the patients" (Participant 20). "I learnt that once I become an authority myself and enter into the hospital environment out of the university campus; I should be more patient, especially with difficult patients" (Participant 18). "Learning humility" was one of the other elements of professional ethics from the perspective of the participants, and its necessity in nursing was highlighted:

"Someday, we were being trained in the Coronary Care Unit (CCU). Our nurse educator told one of the male students: ‘Help the patient eat his breakfast’. The student answered: ‘It is not my nursing duty to do that’. The nurse educator went to the patient and helped him with humility have his breakfast and told: ‘Think that he is your own father hospitalized here’" (Participant 14). "Learning altruism" was another aspect of professional ethics that the nursing students learned and utilized it in the clinical environment: 

"Regardless of their position, the university personnel had an altruism sense toward us and fostered it within us. The sense of altruism was really something beyond the university lessons and I could see how important it is in care" (Participant 10).

#### 4.1.2. Learning Patient-Centeredness

"Learning patient-centeredness" was regarded by the students as one of the other professionalism elements. It represents their endeavor for developing their professional dimension. Here, the students talked about "understanding a patient-centered care" in the hidden curriculum, and stated that they have learnt that patient-centeredness is one of the important and effective factors in care: 

"I know that the patient is afraid of something or is anxious, I shouldn't say: ‘Well I don't care’. I'd rather ask for the reason why. If I could, I would remove it, and if I could do nothing, I would pass it onto a consultant. That is, I have learnt to highlight this matter. I have learnt it from my nurse educator to have the maximal empathy with the patients" (Participant 22). Another student believes that the patients' concerns should be considered regardless of the fact that it is your duty or not: "When you see a patient is suffering pain, don't ask yourself whether it is your duty to help or not. You have to do whatever you can; since it is only the patient who is important. Patient is always preferred in the treatment. In other words, I think that the treatment team should completely concentrate on the patient" (Participant 19).

The effectiveness of a patient-centered care was described by one of the students as: "I saw that upon seeing the nurse, the patient forgot his pain completely; he knows that somebody pays attention to him, and takes necessary measures for him. Consequently, I noticed that his cooperation and interaction improved. I really felt pleased and satisfied with my major" (Participant 19).

### 4.2. Variety of Influenceability Strategies

This theme represents the strategies used by nursing students in the hidden curriculum in their attempt to achieve some degrees of professionalism. The experiences of the participants show that they have been influenced by various strategies, which can be categorized into two subthemes: "observational learning", and "learning from feedback".

#### 4.2.1. Observational Learning

According to the participants, "observational learning" is among the most important strategies used by students in professionalism:

"In the hospital, and at the college, we observed the way the nurse educators were interacting with each other, with our classmates, and with different patients, and also the way their patience, their work ethic, etc. could be really effective; then we tried to behave accordingly" (Participant 20). "In the clinical environment, naturally I learnt some points from my nurse educators. That is I got what to do through observation. For example, I noticed the way which my nurse educator interacted when he wanted to perform a procedure on the patient" (Participant 18).

#### 4.2.2. Learning From Feedback

The next learning method of professionalism through the hidden curriculum in nursing students was "learning from feedback". Here, after observing the effects and consequences of a particular behavior on the self or on others, the student may be influenced reflectively, and will reinforce or forget it accordingly: "Well, you learn from the feedbacks you receive. When you see the feedback and the effect of what you've done, it will automatically get reinforced in your mind. You learn the consequences of whatever you do" (Participant 17). "I saw how respectful a good nurse was to all patients and even to the door keeper. Everybody respects her and our nurse educators as well. Therefore, I learnt that I should be like them" (Participant 23). 

### 4.3. Influenceability to Various Resources

One of the other main extracted themes was "influenceability to various resources". The participants' experiences in professionalism indicate that, to achieve professionalism, they use various resources in the hidden curriculum such as nurse educators, nurses, physicians, peers, and even patients. This theme includes several subthemes, which are the learning resources of students in professionalism.

#### 4.3.1. Influenceability to a Nurse Educator's Behavior

"Influenceability to a nurse educator's behavior" was one of the most important and the most influential of these resources, and the participants considered it to have a critical role in professionalism. Of course, this kind of influence is different from the direct influences that a nurse educator may usually have through formal education: "We had a nurse educator who behaved very suitably. We had to attend the sessions by the last one. The nurse educator told us: ‘I am responsible to tell you these points, and I have to teach them myself so that you can solve your problems in the classroom’. Well, actually such a firm behavior had a direct influence in the profession" (Participant 19).

#### 4.3.2. Influenceability to the Nurses

As the participating students stated, one of the other resources influential in their learning was "influenceability to the nurses" in the clinical environment. Here again, by observing the behavior of these groups in the clinical environment, the students indirectly learnt points relevant to their profession, which essentially included patient care:

"He trained me how to suture; I told him thank you for your patience and the points you taught me. They were a reciprocal behavior and a mutual respect. I learnt how to respect besides being professional and scientific" (Participant 1). "The points I learnt from the nurses ,were all ethical. When I saw that a nurse with work ethic has a different behavior towards the patients from those who do not have it, I learnt that it's a good thing to have work ethic" (Participant 11).

#### 4.3.3. Influenceability to Physicians

"Influenceability to physicians" was one of the other subthemes of influenceability to various resources. According to the participants, it plays an important role in the professionalism of the nursing undergraduates:

"I loved one of the physicians' behavior in Neonatal Intensive Care Unit. She always used to tell us: ‘You have to be completely careful about your behavior with newborns; you should care that newborns you are working with, are very sensitive, and their immune system is still weak ; they are very vulnerable to respiratory distress. You shouldn't wear one single pair of gloves to work with three different newborns’. She further said: ‘You should work with your conscience’. This really influenced me" (Participant 6). "The patient's urine bag was full. One of my friends wanted to empty that because it was really harassing for the patient, and we knew what complications it might bring about. But a nurse said: ‘No, don't do that; it's a nurse assistant’s duty’. Then we saw that a physician came and emptied the bag. It was really instructive for us. Well, physicians are very ideal in our minds, and ideals are always more influential than those usual individuals" (Participant 11).

#### 4.3.4. Influenceability to Peers

"Influenceability to peers" was another subtheme of "learning resources" through the hidden curriculum:

"I learnt a lot from my friends with whom I went to hospital. Each of them had a particular behavior and special words" (Participant 15). "The different cultures of our peers in the university made it possible for us know how to contact with the patients from various social and cultural contexts. It provided suitably for the way to behave more appropriately with the patients whose cultures and personalities were different from ours, to get along with them better, to come to terms with them, and to mentally understand them better; all these were very effective too" (Participant 14).

#### 4.3.5. Influenceability to Patients

Finally, another learning resource for the students in the hidden curriculum of their professionalism was "patients". The participants' speeches represent their "influenceability to the patients" so that they considered the importance of patients in the provision of learning opportunity as analogous to a book:

"I think each patient is like an unread book. Besides their diseases, their personalities are books that can teach us" (Participant 2). "Another kind of learning is learning from different patients. I learnt from one patient and administered it somewhere else. For example, we could learn how to communicate" (Participant 9).

## 5. Discussion

One of the main themes extracted in this study was "development of understanding the professionalism elements". This theme represents the perception and the description of professionalism elements by nursing students. It also shows their beliefs in achieving these elements through the hidden curriculum. The students stated the professionalism elements to be "professional ethics", and "patient-centeredness", and added that they had learnt them during their education period.

"Professional ethics" was one of the other elements of professionalism as stated by the participants. Ethics is an essential principle in care and an inevitable constituent of nursing. In this study, besides the description of ethics as an element of professionalism, the students said implicitly that the hidden curriculum has a key role in learning professional ethics and its internalization in practice. Similarly, other studies showed that the hidden curriculum is the best way for transferring and teaching the ethical principles (17, 18). Researchers believe that for ethical development in students, curriculum planners should consider both the formal curriculum and the hidden one (19). In nursing education, other than the knowledge and skills necessary in this profession, the values, attitudes and ethical norms should be highlighted and, to this end, the role of the hidden curriculum should be considered.

"Patient-centeredness" was stated by the participants as one of the other elements of professionalism. They believed that they had learnt it through the hidden curriculum, and understood its importance and status in care. Given the central and pivotal position of patient in care, achieving this goal can be very valuable in the process of preparing nursing students. Patient-centeredness is so important in professionalism that some medical and nursing texts mention the concept of patient-centered professionalism (20). Lamiani et al. who investigated the hidden curriculum in Italian medical students, extracted the two major themes of "disease-centered medicine" and "delegation of patients' emotional needs to nurses" (10).

Of the other themes emerged in this study was "variety of influenceability methods". This theme represents the various strategies that students use to self-learn the things relevant to professionalism in the hidden curriculum. These strategies include "observational learning", and "learning from feedback". "Observational learning" has been the main and the simplest method of learning for students in which they maintain their sensitivity towards the ongoing situations and events, and learn and use them via a critical thinking approach. In fact, this approach is superior from a mere imitation of a specified behavior. What students listen to, in the classrooms, does not exercise the most permanent influence, but what they observe from their educators and others, influences their attitudes and understandings of true expectations from the profession. Glicken and Merenstein argue that observation of behaviors in different wards of the hospital by students is considerably more influential than the theoretical material they learn in the classrooms about appropriate behavior (21). In fact, students' behavior with patients, colleagues and their future students is the same as the way the educators behave with them. Students repeat what their educators do, not what they say (22). Puckett et al. reported that students learn better in the environments where they can observe and exercise professional roles (23).

The other learning method through the hidden curriculum was "learning from feedback"; the students claimed that after performing a particular behavior and even after observing specific behaviors in other people, in the case that they are reinforced and given that they have desirable results and positive feedbacks in the environment; such behaviors will be utilized in similar situations and will become a part of their professional personality. There are different studies on the influence of feedback in formal educational programs, and its positive influences on teaching different behaviors which have been proven for all ages. However, there are few studies on the process of feedback and people's influenceability to feedbacks in the environment of hidden curriculum. Yet the findings of this study showed that regardless of its being formal or informal, purposeful or non-purposeful feedback, can be effective in the reinforcement and establishment of a particular behavior. Researchers argue that feedback is an essential dimension of teaching and learning, and is considered a "critical force of learning". The importance of feedback has been proven, especially in clinical teaching (24).

"Influenceability to various resources" was another theme achieved in this study. The students used different learning resources for learning professionalism, each of which had important impacts on the students. They included: nurse educators, nurses, physicians, peers and patients. Students' influenceability to these resources in the hidden curriculum can also be described within the concept of "role models", because it can be inferred from the participants' words that they moved toward achieving professional competencies mostly through observation of their behaviors and modeling them. The importance of role models in the professionalism of nursing students is well supported (25). The results of Stern’s study on the hidden curriculum in the hospital wards with regards to value transfer, showed that the degree of the values that the students learnt, was higher when the medical teachers were present than when they were absent. In addition, values are mostly learnt through the hidden curriculum than through the formal curriculum (26). One of the other role models in this study was "nurses". Nurses' practices are observed in the clinical environment. Patients, family members and other nurses observe their actions and interactions. Nurses are often potential role models who can have both negative and positive influences on the behaviors and attitudes of others (27). Role models in nursing are important in the clinical learning environment (28). "Physicians" was of other learning resources in professionalism through the hidden curriculum for the nursing students in this study. Physicians and medical teachers, as the major members of health care team, always play a significant role in the clinical environment. Moreover, students regard them as the ideal clinical role models. Therefore, their behavior can influence the students and provide for their learning. "Peers" was one of the resources of learning through the hidden curriculum in the present study. Karnieli-Miller et al. also found peers as one of the learning resources in professionalism through the hidden curriculum (29). According to Salehi, the majority of students believed that the instructors in the clinical wards were their main sources of hidden learning. However, behaviors of the staff, as well as their peers were considered important (30). And finally, the participants of this study introduced "patients" as another learning resource in professionalism. So far, there has been little consideration of the role of patients as a learning resource through the hidden curriculum in professionalism. Nevertheless, the clinical nature of nurse training per se indicates the importance of patients in learning because here students play an actual role and their learning will become more permanent. It can be used as a learning resource in the clinical educational programs of educators.

### 5.1. Conclusions

According to the view of nursing undergraduates in this research, the elements of professionalism were "professional ethics" and "patient- centeredness", which are to be learnt through the hidden curriculum using different influenceability methods including "observational learning", and "learning from feedback", and are influenced by nurse educators, nurses, physicians, peers and patients. Therefore, the hidden curriculum being practiced, should be explored exactly and permanently, and strategies should be specified for encountering with its negative outcomes and reinforcing its positive outcomes. Consequently, because of the importance of the hidden curriculum in learning the nursing professionalism, specifying the hidden factors (which are of social use) and presenting them in the explicit curriculum can prevent the hidden curriculum from residing in the black box. Furthermore, since nurse educators and nurses can act as role models, they should possess these attributes themselves in advance. Furthermore, the importance of role modeling of physicians in teaching nursing professionalism should be highlighted more.

### 5.2. Limitations

Similar to other qualitative studies, caution should be exercised in generalizing the results of this study because of the limitations inherent in it. It is natural that the results of this study will be used only in the studied population, and its application in other communities needs further studies. However, with regard to the similarity of the nursing students’ characteristics, and also the nursing education programs throughout the country, these findings are applicable to the community of nursing students in Iran.

## References

[1] Finkelman AW, Kenner C (2013). Professional Nursing Concepts: Competencies for Quality Leadership..

[2] Kim CJ, Ahn YH, Kim MW, Jeong YO, Lee JH (2006). [Development of standards and criteria for accreditation of a baccalaureate nursing education program: reflections on the unique characteristics of the nursing profession].. Taehan Kanho Hakhoe Chi..

[3] Kim-Godwin YS, Baek HC, Wynd CA (2010). Factors influencing professionalism in nursing among Korean American registered nurses.. J Prof Nurs..

[4] Primm RD (2010). Professionalism among occupational health nurses.. Aaohn J..

[5] Stern DT, Papadakis M (2006). The developing physician--becoming a professional.. N Engl J Med..

[6] Holosko M, Skinner J, MacCaughelty C, Stahl KM (2010). Building the implicit BSW curriculum at a large Southern state university.. J Soc Work Educ..

[7] Gaiser RR (2009). The teaching of professionalism during residency: why it is failing and a suggestion to improve its success.. Anesth Analg..

[8] Kommalage M (2011). Hidden and informal curricula in medical schools: impact on the medical profession in Sri Lanka.. Ceylon Med J..

[9] Tabari Khomeiran R, Deans C (2007). Nursing education in Iran: past, present, and future.. Nurse Educ Today..

[10] Lamiani G, Leone D, Meyer EC, Moja EA (2011). How Italian students learn to become physicians: a qualitative study of the hidden curriculum.. Med Teach..

[11] Balmer DF, Master CL, Richards B, Giardino AP (2009). Implicit versus explicit curricula in general pediatrics education: is there a convergence?. Pediatrics..

[12] Speziale HS, Carpenter DR (2007). Qualitative Research in Nursing: Advancing the Humanistic Imperative..

[13] Holloway I (2008). A-Z of Qualitative Research in Nursing and Healthcare..

[14] Polit DF, Beck CT (2013). Essentials of Nursing Research: Appraising Evidence for Nursing Practice..

[15] Graneheim UH, Lundman B (2004). Qualitative content analysis in nursing research: concepts, procedures and measures to achieve trustworthiness.. Nurse Educ Today..

[16] Lincoln YS, Guba EG (1985). Naturalistic Inquiry..

[17] Hafferty FW, Franks R (1994). The hidden curriculum, ethics teaching, and the structure of medical education.. Acad Med..

[18] Howard F, McKneally MF, Upshur RE, Levin AV (2012). The formal and informal surgical ethics curriculum: views of resident and staff surgeons in Toronto.. Am J Surg..

[19] Goldie J (2000). Review of ethics curricula in undergraduate medical education.. Med Educ..

[20] Hutchings H, Rapport F, Wright S, Doel M, Jones A (2012). Obtaining consensus about patient-centred professionalism in community nursing: nominal group work activity with professionals and the public.. J Adv Nurs..

[21] Glicken AD, Merenstein GB (2007). Addressing the hidden curriculum: understanding educator professionalism.. Med Teach..

[22] Kassebaum DG, Cutler ER (1998). On the culture of student abuse in medical school.. Acad Med..

[23] Puckett AC, Graham DG, Pounds LA, Nash FT (1989). The Duke University program for integrating ethics and human values into medical education.. Acad Med..

[24] Clynes MP, Raftery SE (2008). Feedback: an essential element of student learning in clinical practice.. Nurse Educ Pract..

[25] Livsey KR (2009). Structural empowerment and professional nursing practice behaviors of baccalaureate nursing students in clinical learning environments.. Int J Nurs Educ Scholarsh..

[26] Stern DT (1998). Practicing what we preach? An analysis of the curriculum of values in medical education.. Am J Med..

[27] Perry RN (2009). Role modeling excellence in clinical nursing practice.. Nurse Educ Pract..

[28] Donaldson JH, Carter D (2005). The value of role modelling: Perceptions of undergraduate and diploma nursing (adult) students.. Nurse Educ Pract..

[29] Karnieli-Miller O, Vu TR, Frankel RM, Holtman MC, Clyman SG, Hui SL (2011). Which experiences in the hidden curriculum teach students about professionalism?. Acad Med..

[30] Salehi S (2006). Students’ Experience with the Hidden Curriculum in the Faculty of Nursing and Midwifery of Isfahan University of Medical Sciences.. J Med Educ..

